# Accuracy and the factors influencing the accuracy of death certificates completed by first-year general practitioners in Thailand

**DOI:** 10.1186/s12913-018-3289-1

**Published:** 2018-06-20

**Authors:** Chaiwat Washirasaksiri, Prateep Raksasagulwong, Charoen Chouriyagune, Pochamana Phisalprapa, Weerachai Srivanichakorn

**Affiliations:** 1 Division of Ambulatory Medicine, Department of Medicine, Faculty of Medicine Siriraj Hospital, Mahidol University, 2 Wanglang Road, Bangkoknoi, Bangkok, 10700 Thailand; 2 Department of Medicine, Faculty of Medicine Siriraj Hospital, Mahidol University, 2 Wanglang Road, Bangkoknoi, Bangkok, 10700 Thailand

**Keywords:** Thailand, Death certificate, General practitioner, GP, Quality, Influencing factors

## Abstract

**Background:**

Although death certificates (DCs) provide valuable health information which may help to guide local health policies and priorities, there is little information concerning their validity in Thailand. First-year general practitioners (GPs) have a major role in DC completion, especially in provincial general hospitals. The aim of this study was to evaluate the accuracy and factors influencing the accuracy of DCs completed by first-year GPs in Thailand, compared with the cause of death (COD) derived from medical records by experts.

**Methods:**

This retrospective study was conducted at 14 provincial general hospitals in Thailand during the June 2011 to May 2012 study period. Medical records and DCs completed by first-year GPs who graduated from 16 Thai medical schools were sampled. The cause of death recorded on the DCs was compared with the medical conditions and histories derived from patient medical records. A cross-sectional survey of the 210 GPs who completed the DCs reviewed in this study was also conducted. Respondent GPs’ demographic characteristics, factors associated with COD, and COD coding system were evaluated.

**Results:**

Five hundred and sixty-three medical records and corresponding DCs were included. Of those, 36.9% of DCs were found to be correct. Common mistakes included incorrect sequence of events leading to death (32.4%), and mode of death use (26.2%). Of the 210 GPs, 155 questionnaires were completed and returned. The mean time spent on recording COD and completing DCs in the medical school curriculum was 2.1 ± 0.9 h and only 27.7% of participants had experience in completing DCs by themselves during medical school. Mean medical school GPA was significantly higher in the correctly completed DC GPs group than in the incorrectly completed DC GPs group (3.3 ± 0.4 vs. 3.2 ± 0.3; *p* = 0.03). However, no significant difference was found for other factors associated with COD between groups.

**Conclusions:**

This is the first study documenting gaps and disparities in DC accuracy, and factors influencing completion of DCs among first-year GPs in Thailand, based on a clinical assessment of medical records. GPs made errors on 63.1% of DCs. This finding suggests that proven education, system-related support, and additional training interventions specific to DC completion are required.

**Electronic supplementary material:**

The online version of this article (10.1186/s12913-018-3289-1) contains supplementary material, which is available to authorized users.

## Background

Death certificates (DCs) provide valuable national health information regarding population-based mortality, disease incidence, and disease prevalence – particularly in some preventable diseases. This information may lead to the development and implementation of local health policy, and help to guide resource prioritization and management [1]. DCs provide valuable data for health researchers and epidemiological studies, and also benefit families by providing data about risk factors for inherited diseases. Although DCs are an important tool for reporting and gathering vital statistics within a population, there is little evidence concerning their validity and the factors that influence their accuracy [1–4]. Death certificate (DC) errors contribute to unreliable results in population-based studies and inaccurate public health policy trends [5]. Previous reviews have demonstrated that DC completion errors are common [6–9], ranging from 25.0 to 78.0% in a hospital-based study [7, 8]. In terms of a gold standard, there have been different approaches to assessing DC accuracy, including autopsy data, verbal autopsy, and the use of medical records [10–14].

A previous study [15] reported that recent mortality statistics in Thailand were of low quality, with over 30.0% of deaths unregistered and more than 20.0% of underlying causes of death (COD) classified as “ill-defined cause”. In Thailand, first-year general practitioners (GPs) have a major role in DC completion, especially in provincial general hospitals. Although this is a multi-factorial problem [16]*,* one of the possible explanations is that some physicians may not receive sufficient training and support in DC completion, and this may result in inaccurate in the completion and recording of inaccurate DCs [17]. The aim of this study was to investigate the accuracy of DCs, and to identify patterns and characteristics of DC errors. Physician-related factors, such as demographics, knowledge, experience, DC education, support systems, and other possible factors that may influence DC completion, were also evaluated.

## Methods

This retrospective study was conducted at 14 provincial general hospitals in Thailand during the June 2011 to May 2012 study period. First-year general practitioners who graduated from 16 public medicals school registered with the Medical Council of Thailand in 2011 were randomly selected to receive questionnaires. These 16 medical schools produce approximately 2000 medical graduates each year. Medical graduates from private and unregistered medical schools were excluded. A total of 210 first-year general practitioners from 16 medical schools were randomly selected from 14 of 50 (28.0%) provincial general hospitals from all regions of Thailand. Approximately 10.0% of the total number of GPs from each medical school was included in this study. Included GPs had to have a minimum of 3 charts and 3 associated DCs. The quality of the charts was assessed and incomplete medical records were excluded. A cross-sectional questionnaire was distributed to randomly selected physicians to explore factors, such as knowledge about DCs, education, workload, and experience in completing DCs.

The DC forms used to report the underlying COD in Thai hospitals follow World Health Organization guidelines [18]. Information is recorded in a three-part format. The sequence of events leading to the COD are recorded in part one. Part two is used to record other significant conditions contributing to death. Part three, which is only used in Thai DCs, is a section translated into Thai. The COD is defined as the disease or injury that initiated the train of morbid events directly leading to death, or the circumstances of the accident or violence that produced the fatal injury. The COD is recorded on the last line of part one. The present study evaluated the recorded COD and translation section of all included DCs. The accuracy of this information was corroborated and validated by reviewing the relevant information in the corresponding medical records. The initial evaluation was conducted by one trained doctor and one auditor. In case of discrepancies, a third expert made an independent judgment, with final agreement reached via consensus. Correct COD was defined as both selected parts of the DC being correct according to the judgement of the reviewers. Participants who coded an appropriate COD in more than 60% of the DCs they completed were classified as the “correct DC GP group”, with the remaining participants classified as the “incorrect DC GP group.” Common mistakes on the death certificate were classified and defined as follows: 1) *Incorrect sequence of events leading to death –* an incorrect temporal ordering of information in the part 1 cascade; 2) *Mode of death use* – an incorrect selection of the manner of death, such as respiratory failure or cardiac arrest, in the part 1 cascade; 3) *Nonspecific cause of death* – giving a generic cause of death (e.g., sepsis), which, though a disease process, was then not followed-up with an underlying cause of death; 4) *Linkage errors* – selecting a cause of death that is linked by a provision in the classification; 5) *Incorrect COD recorded in the comorbidity section* that is transposed from part 1 (the cause of death) to part 2 (contributory conditions) or vice versa; 6) *Incomplete diagnosis* or no COD on the DC; 7) *Errors in Thai translation*; and, 8) *Trivial condition*– the selected cause is a minor condition unlikely to cause death.

Clinical parameters associated with DC accuracy, including physician-specific factors and COD coding systems were collected. Data collected by questionnaire included demographic characteristics (age, gender, medical school grade point average [GPA], workload, hospital at which the participant works, education, and future career/medical specialization plans); factors associated with COD accuracy (DC knowledge, confidence in completing DCs, and factors influencing errors); and COD coding systems (training in COD coding, responsible person).

### Sample size calculation and statistical analysis

The proportion of general internal medical residents completing correct death certificates was found to be 65.0% from a previous survey conducted at the Department of Medicine, Faculty of Medicine Siriraj Hospital. The estimate of the proportion of the population by setting the relative error tolerance was 10% of the proportion of medical graduates completing correct death certificates at 95% confidence level. The calculation of the sample size used in this study, approximately 210 first-year general physicians should be recruited. The minimum of 3 charts and 3 associated DCs per each physician were required for evaluation. Therefore, 630 charts and associated DCs were recruited in this present study.

Completed questionnaires, DCs, and medical records were assessed. Categorical data are presented as frequencies and percentages. Categorical data, such as factors associated with the accuracy of COD, were compared using chi-square test or Fisher’s exact test if the element was an enumeration data point. Continuous data are expressed as mean ± standard deviation (SD), with *t*-test used to compare differences between groups. Odds ratios of parameters of accuracy of DC completion between the correct and incorrect DC groups and odds ratios of each disease in correctly completed DCs were assessed using univariate logistic regression analysis. All statistical analyses were performed using SPSS Statistics version 13.0 (SPSS, Inc., Chicago, IL, USA). For all analyses, a *p*-value of less than 0.05 was considered statistically significant.

## Results

### Medical records and DCs

In total, 622 medical records and corresponding DCs were obtained from 14 provincial general hospitals in Thailand. Of those, 59 medical records and DCs were excluded, because certain aspects of those records were inaccessible or incomplete. In the end, 563 medical records and corresponding DCs were enrolled and included in the final analysis. The mean age of deceased patients was 58.0 ± 21.5 years and 45.7% of them were female. The predominant cause of death (125 patients, 22.2%) was cardiovascular disease. Only 36.9% (*n* = 208) of DCs were correct for both identification of the events that led to the patient’s death and COD coding. Common mistakes found on incorrectly completed DCs included incorrect sequence of events leading to death (32.4%), mode of death use (26.2%), nonspecific cause of death (25.7%), linkage errors (22.3%), incorrect COD recorded in the comorbidity section (13.9%), incomplete diagnosis (7.1%), no COD on the DC (6.6%), errors in the Thai translation (6.4%), and trivial condition (0.7%). In addition, there were errors on the DCs concerning the leading groups of diseases, such as endocrine diseases (93.0%), musculoskeletal and rheumatologic disease (90.0%), infectious diseases (80.2%), neurological diseases (78.6%), and external causes (75.0%). However, 31.7% of the correctly completed DCs reported cardiovascular disease and 24.5% reported cancer; whereas, only 16.6% of DCs with errors reported cardiovascular disease and 8.2% reported cancer. The odds ratio (OR) for cardiovascular disease in the correctly completed DCs was 2.3 (95% confidence interval [CI]: 1.6–3.5; *p* < 0.001), and for cancer the OR was 3.7 (95% CI: 2.2–6.0; *p* < 0.001). In contrast, 18.3% of the DCs with errors reported infectious diseases and 11.3% reported endocrine diseases; whereas, only 7.7% of all correctly completed DCs reported infectious diseases (OR: 0.4, 95% CI: 0.2–0.7; *p* = 0.001) and 1.4% reported endocrine diseases (OR: 0.1, 95% CI: 0.04–0.4; *p* < 0.001). Death certificate characteristics compared between the correctly and incorrectly completed DC groups and odds ratio of predictors of prevalence of correctly completed DCs are shown in Tables 1 and 4.

**Table 1:** Death certificate and medical record data among groups

Death certificate characteristics	All DCs (*n* = 563)	Correct DCs (*n* = 208)	Incorrect DCs (*n* = 355)
1. Age (yr), mean ± SD	58.0 ± 21.5	60.2 ± 20.6	56.7 ± 22.0
2. Female gender: *n* (%)	207 (45.7)	84 (47.5)	123 (44.6)
3. Evaluation of events leading directly to COD, *n* (%)	563 (100)	321 (57.0)	242 (43.0)
4. Thai COD coding evaluation, *n* (%)	563 (100)	229 (40.7)	333 (59.3)
5. Hospital size: *n* (%)			
▪ > 1000 beds	118 (21.0)	43 (20.7)	75 (21.1)
▪ 701–1000 beds	160 (28.4)	67 (32.2)	93 (26.2)
▪ 401–700 beds	285 (50.6)	98 (47.1)	187 (52.7)
6. Hospital location by Thailand region, *n* (%)			
▪ Northeastern	178 (31.6)	61 (29.3)	117 (33.0)
▪ Northern	105 (18.7)	39 (18.8)	66 (18.6)
▪ Southern	104 (18.5)	44 (21.2)	60 (16.9)
▪ Eastern	71 (12.6)	25 (12.0)	46 (13.0)
▪ Central	105 (18.7)	39 (18.8)	66 (18.6)
7. Disease group, *n* (%)			
▪ Cardiovascular disease	125 (22.2)	66 (31.7)*	59 (16.6)*
▪ Infectious disease	81 (14.4)	16 (7.7)**	65 (18.3)**
▪ Cancer disease	80 (14.2)	51 (24.5)*	29 (8.2)*
▪ Gastrointestinal disease	77 (13.7)	27 (13.0)	50 (14.1)
▪ Pulmonary disease	53 (9.4)	22 (10.6)	31 (8.7)
▪ Endocrine disease	43 (7.6)	3 (1.4)*	40 (11.3)*
▪ External cause	36 (64.0)	9 (4.3)	27 (7.6)
▪ Nephrology disease	18 (3.2)	5 (2.4)	13 (3.7)
▪ Neurologic disease	14 (2.5)	3 (1.4)	11 (3.1)
▪ Musculoskeletal and rheumatologic disease	10 (1.8)	1 (0.5)	9 (2.5)
▪ Unknown cause of death	8 (2.3)	0 (0)	8 (2.3)
▪ Other	18 (3.2)	5 (2.4)	13 (3.7)

Categorical variable; number (percent), continuous normally-distributed variable; mean ± standard deviation*.* Significant differences across correct and incorrect death certificate categories were identified for normally distributed continuous variables by t-test,

Abbreviations: *N* Number, *%* Percent, *COD* Cause of death, *SD* Standard deviation, *yr*. Year

*p*-value < 0.05 indicates statistical significance; *p < 0.001; **p = 0.001

### Questionnaire responses from first-year general practitioners

One hundred eighty-nine (189) of 210 (response rate 90%) surveys were completed and returned. Of those, 34 questionnaires were excluded because the clinical records of their deceased patients were incomplete, not available, or the questionnaires were completed by physicians who completed a DC for less than three deceased patients enrolled in this study. The remaining 155 questionnaires were included for analysis. The mean age of physician respondents was 25.3 ± 0.8 years, and 56.8% were female. The mean medical school GPA was 3.2 ± 0.3. Just over three quarters (76.2%) of respondents graduated from large medical schools (defined as producing more than 100 physicians per year). Of 155 physicians, 47 (30.3%; range for each medical school: 21.4–50.0%) were able to correctly complete more than 60% of DCs (correct DC group). The remaining 108 physician respondents (69.7%) showed poor performance in DC completion (incorrect DC group). Mean medical school GPA was significantly higher in the correctly completed DC group than in the incorrectly completed DC group (3.3 ± 0.4 vs. 3.2 ± 0.3; *p* = 0.03). The odds ratio for accuracy of DCs in participants with a high GPA (GPA ≥3.5) was 3.5 (95% CI: 1.5–7.9; *p* = 0.003). However, the other characteristics were not significant different between groups. Characteristics of first-year general practitioners by correct and incorrect death certificate groups and odds ratio of predictors of prevalence of correctly completed DCs are shown in Tables 2 and 4. Questionnaire for evaluation of cause of death summary is shown in Additional file 1.

**Table 2:** Characteristics of first-year general practitioners by correct and incorrect death certificate groups

Characteristics	All general practitioners (*n* = 155)	Correct DC GP group (*n* = 47)	Incorrect DC GP group (*n* = 108)
1. Age (yr.), mean ± SD	25.3 ± 0.8	25.2 ± 0.7	25.4 ± 0.8
2. Female gender, *n* (%)	88 (56.8)	23 (48.9)	65 (60.2)
3. GPA, mean ± SD	3.2 ± 0.3	3.3 ± 0.4*	3.2 ± 0.3*
4. Hospital size (beds), mean ± SD	820.5 ± 317.5	844.6 ± 342.1	810.0 ± 307.3
5. Workload, mean ± SD			
▪ Number of OPD patients/day	37.2 ± 17.8	37.9 ± 19.5	36.9 ± 17.2
▪ Number of IPD patients/day	32.9 ± 18.8	32.6 ± 14.2	33.0 ± 20.4
▪ Number of IPD patients on night duty/day	17.9 ± 11.4	17.2 ± 10.6	18.2 ± 11.7
6. Size of medical school graduating class, *n* (%)			
▪ > 200 medical graduates	56 (36.2)	18 (38.3)	38 (35.2)
▪ 101–200 medical graduates	62 (40.0)	18 (38.3)	44 (40.7)
▪ 51–100 medical graduates	21 (13.5)	4 (8.5)	17 (15.7)
▪ < 50 medical graduates	16 (10.3)	7 (14.9)	9 (8.3)
7. Future plans for specialist training, *n* (%)			
▪ No future training plans	30 (19.4)	8 (17.0)	22 (20.4)
▪ Major field training (i.e. medicine, surgery, pediatrics, obstetrics and gynecology)	88 (56.8)	27 (57.4)	61 (56.5)
▪ Minor field training	37 (23.9)	12 (25.5)	25 (23.1)

Categorical variable; number (percent), continuous normally-distributed variable; mean ± standard deviation*.* Significant differences across correct and incorrect DC GP group were identified for normally distributed continuous variables by t-test

Abbreviations: *N* Number, *%* Percent, *DC* Death certificate, *GP* General practitioner, *SD* Standard deviation, *yr.* Year, *OPD* Outpatient department, *IPD* Inpatient department, *SD* Standard deviation, *GPA* Grade point average

*p*-value < 0.05 indicates statistical significance; * *p* = 0.03

### Clinical parameters associated with accurately completed DCs

In terms of physician-related factors, the mean time spent on recording COD and completing DCs in the medical school curriculum was 2.1 ± 0.9 h, and 73.8% of participants reported spending less than 2 h. In addition, only 27.7% of participants had experience in completing DCs by themselves during medical school. The questionnaire responses indicated that 41.9% of participants had adequate knowledge of COD coding. Only 42.6% of respondents were aware that DCs are used as health indicators and monitoring tools for public health policy. Only 25.2% of participants could differentiate between COD and mode of death (MOD), and 26.5% reported being confident in completing DCs. The physicians that participated in this study reported being responsible for 81.8% of COD coding on DCs during office hours, and 99.6% of COD coding on DCs after hours. Only 10.4% of participants reported that official staff took this responsibility and provided supervision during COD coding. In addition, only 29.0% of the hospitals had established DC training courses for new doctors. Common reported factors associated with DC completion were lack of clinical knowledge (32.9%). However, these factors did not differ between the correct DC and incorrect DC groups. Clinical parameters associated with correct death certificates completed by first-year general practitioners between groups and odds ratio of predictors of prevalence of correctly completed DCs are presented in Tables 3 and 4.

**Table 3:** Clinical parameters associated with death certificates completed by first-year general practitioners

Clinical parameters	All general practitioners (*n* = 155)	Correct DC GP group (*n* = 47)	Incorrect DC GP group (*n* = 108)
1. Overall DC knowledge, *n* (%)	65 (41.9)	21 (44.7)	44 (40.7)
▪ Understands that DCs are used in public health research and policy, *n* (%)	66 (42.6)	23 (48.9)	43 (39.8)
▪ Can differentiate between COD and MOD, *n* (%)	39 (25.2)	14 (29.8)	25 (23.1)
2. High level of self-confidence in identifying correct underlying COD, *n* (%)	41 (26.5)	15 (31.9)	26 (24.1)
3. Factors influencing errors in COD, *n* (%)			
▪ Lack of appropriate knowledge	51 (32.9)	15 (31.9)	36 (33.3)
▪ Extenuating circumstance^a^	17 (11.0)	3 (6.4)	14 (13.0)
▪ High workload	33 (21.3)	7 (14.9)	26 (24.1)
▪ Lack of adequate data	28 (18.1)	10 (21.3)	18 (16.7)
▪ Other/non applicable	26 (16.7)	12 (25.5)	14 (12.9)
4. Time spent on COD determination and DC completion in medical curriculum (hr), mean ± SD	2.1 ± 0.9	2.2 ± 0.7	1.8 ± 0.9
▪ Time spent on COD and DCs < 2 h, *n* (%)	96 (73.8)	29 (74.4)	67 (73.6)
5. COD coding experience, *n* (%)			
▪ Have experience completing DCs by themselves during medical school	43 (27.7)	17 (36.2)	26 (24.1)
▪ Have experience completing DCs by themselves > 5 cases/month after graduation	105 (67.7)	33 (70.2)	72 (66.7)
6. Established COD coding training program in work hospital, *n* (%)	45 (29.0)	13 (27.7)	32 (29.6)
7. Person responsible for completion of death certificates during office hours, *n* (%)			
▪ Primarily senior physicians	16 (10.4)	5 (10.6)	11 (10.3)
▪ Primarily general practitioners	126 (81.8)	38 (80.9)	88 (82.2)
▪ On-duty general practitioner	3 (2.0)	0 (0)	3 (2.8)
▪ Other	9 (5.8)	4 (8.5)	5 (4.7)
8. Person responsible for completion of death certificates during after hours, *n* (%)			
▪ Primary senior physicians	3 (2.0)	1 (2.1)	2 (1.9)
▪ Primary general practitioners	31 (20.1)	16 (34.0)	15 (14.0)
▪ On-duty general practitioner	112 (72.7)	27 (57.5)	85 (79.4)
▪ Other	8 (5.2)	3 (6.4)	5 (4.7)

Categorical variable; number (percent), continuous normally-distributed variable; mean ± standard deviation*.* Significant differences across correct and incorrect DC GP group were identified for normally distributed continuous variables by t-test

Abbreviations: *N* Number, % Percent, *COD* Cause of death, *MOD* Mode of death, *DC* Death certificate, *DCs* Death certificates, *SD* Standard deviation, *GP* General practitioner

*p*-value < 0.05 indicates statistical significance; all parameters in this table were *p* above 0.05

^a^An example of an extenuating circumstance would be a family member of the deceased that requests that you alter the COD in order to conceal the fact that the patient had HIV infection

**Table 4:** Predictors of prevalence of correctly completed death certificates: Logistic regression analysis, univariate analysis

Death certificate characteristics	Unadjusted OR (95% CI)	*p* value	GP characteristics and associated parameters.	Unadjusted OR (95% CI)	*p* value
Elderly (age ≥ 60 yr.)	1.2 (0.8–1.8)	0.2	Age	0.8 (0.5–1.3)	0.3
Female	1.1 (0.8–1.6)	0.5	Female	0.6 (0.3–1.3)	0.1
Hospital Sizes			Medical School Sizes		
▪ 400–700 beds	Reference		▪ > 200 graduates/ year	Reference	
▪ 701–1000 beds	1.1 (0.7–1.7)	0.6	▪ 101–200 graduates/ year	0.9 (0.4–1.9)	0.7
▪ More than 1000	1.4 (0.9–2.0)	0.1	▪ 51–100 graduates/ year	0.5 (0.2–1.7)	0.2
COD disease groups			▪ ≤ 50 graduates/ year	1.6 (0.5–5.1)	0.3
▪ Cardiovascular disease	2.3 (1.6–3.5)	< 0.001	Future plans for specialist training		
▪ Infectious disease	0.4 (0.2–0.7)	0.001	▪ Specialist training plans	1.3 (0.5–3.1)	0.6
▪ Cancer group	3.7 (2.2–6.0)	< 0.001	GPA Group		
▪ Gastrointestinal disease	0.9 (0.6–1.5)	0.7	▪ GPA ≥ 3.5	3.5 (1.5–7.9)	0.003
▪ Pulmonary disease	1.2 (0.7–2.2)	0.4	Time spent during medical curriculum		
▪ Endocrine disease	0.1 (0.04–0.4)	< 0.001	▪ Time spent ≥2 h	1.0 (0.4–2.3)	0.9
▪ External cause	0.6 (0.3–1.2)	0.1	COD coding experience		
▪ Nephrology disease	0.7 (0.2–1.8)	0.4	▪ Have experience in completing DCs by themselves during medical school	1.8 (0.9–3.8)	0.1
▪ Neurologic disease	0.5 (0.1–1.7)	0.2	▪ Have experience completing DCs by themselves >5cases/month after graduation	1.2 (0.6–2.5)	0.6
▪ Musculoskeletal and rheumatologic disease	0.2 (0.02–1.5)	0.07	▪ Established COD coding training program in work hospital	0.9 (0.4–1.9)	0.8
▪ Unknown cause of death/ Other	0.4 (0.2–1.1)	0.05	Understands that DCs are used in public health research and policy	1.5 (0.7–2.9)	0.2

Abbreviations: *DCs* Death certificates, *OR* Odds ratio, *CI* Confidence interval, *COD* Cause of death, *DC* Death certificate, *DCs* Death certificates, *GP* General practitioner, *GPA* Grade point average, *yr.* year

## Discussions

Unreliable COD data can contribute to misleading appraisals of research and poor implementation of health-related activity. Therefore, the evaluation of DC completion accuracy rates, error patterns, and relevant influencing factors is necessary to improve these data. To our knowledge, this is the first study to document the quality of COD coding in Thai hospital-based DCs completed by first-year general practitioners. We also investigated the parameters influencing accurate DC completion of these physicians graduated from a majority of medical schools in Thailand. Only 36.9% of DCs had accurately coded COD, when compared with case data in medical records. The correct DC completion result found in this study was lower than rates reported in other countries, including Canada (67.1%) [19], Australia (84.0%) [20], and the UK (41.4–68.0%) [8, 21, 22]. In addition, only 30.3% of the participating first-year physicians demonstrated good performance in DC completion. A previous study in the US [23] found that 23.0% of DCs completed by physicians were of poor overall quality. Overall, we found higher rates of common COD coding error patterns than were found in a previous US study [17], including incorrect order (32.4% vs. 3.5%), mode of death (26.2% vs. 4.4%), nonspecific COD (25.7% vs. 14.8%), and incorrectly completed DCs (13.9% vs. 6.5%), all respectively. However, the US study was conducted in a suburban community in Broward County, Florida, and deceased patient medical records were not assessed. We found that the COD most commonly coded appropriately was malignancy of any type, followed by cardiovascular disease; whereas, accuracy of DC completion was low for endocrine and infectious diseases. This finding was consistent with two previous studies [14, 24].

Previous research in various countries attributed the validity of DCs to a number of factors relating to quality of undergraduate and postgraduate training [25], patient characteristics (age, sex) and disease responsible for the patient’s death [26], type and size of hospital, and legislation governing death certification [25]. In the present study, we found that only 21.4 to 50.0% of students who graduated from each Thai medical school demonstrated good performance on DC completion. A higher GPA was the only physician-specific factor associated with the accuracy of DC completion. Although other relevant factors were not significantly different between GPs who completed DCs incorrectly and those who completed them correctly, we found that little training and little coding experience was reported by almost all students. Time spent learning about and gaining experience in DCs in the medical curriculum was low, and there was a lack of support systems or coaching in accurate COD coding. In addition, less than half of the first-year physicians reported having adequate knowledge, awareness, and self-confidence about COD coding. Correspondingly, correct methods for DC completion were briefly encountered in medical school, but studies that reviewed DC accuracy [24, 27] reported a significant need for more education. These indicate a need for more training and a more concerted effort to heighten the awareness of physicians regarding the importance of COD determination, and DC completion and coding in Thailand. DC-related skill improvement interventions have included a training package [25], workshops [28], professional development activities, and published materials [29] were required. Lastly, a recent systematic review [30] confirmed that education, especially in an interactive format, and feedback for certifiers of death should be recognized as the principal requirement for high-quality mortality statistics.

## Conclusions

Although DC and COD data are used worldwide for epidemiology, research, and public health policy, the results of the present study reveal some inaccuracy in death-related documents completed by first-year general practitioners in Thailand. Moreover, this is the first study to document gaps in DC accuracy and the factors that influence DC accuracy among physicians, in terms of their understanding of death certification based on clinical assessment of medical records. In Thailand, this is most likely due to a lack of adequate training in the medical school curriculum and a lack of workplace-based support and coaching in provincial general hospitals. Accordingly, proven education, system-related support, and additional training interventions to improve the accuracy of DC completion are needed.

## Additional file

Additional file 1: Questionnaire for evaluation of cause of death summary. (DOCX 20 kb)

## Abbreviations

CI: Confidence interval
COD: Cause of death
DC: Death certificate
DCs: Death certificates
GP: General practitioner
GPA: Grade point average
GPs: General practitioners
OR: Odds ratio
SD: Standard deviation
