# Supplementary material for: Accuracy and the factors influencing the accuracy of death certificates completed by first-year general practitioners in Thailand
Source: BMC Health Serv Res. 2018 Jun 20;18:478. doi: 10.1186/s12913-018-3289-1 (PMC6011513; doi:10.1186/s12913-018-3289-1)
Supplement: Supplementary file 1 — Questionnaire for evaluation of cause of death summary. (DOCX 20 kb) [file 12913_2018_3289_MOESM1_ESM.docx]

**Questionnaire for evaluation of cause of death summary**

**Part 1: Personal and work information**

**Gender:** □ male □ female **Age:** years (year of birth………………)

**Number of hospital beds at your center: __________**

**Your cumulative medical school GPA was** _ _ _ _ .

**Your future medical field/career interest** **is** .

**Workload**

The number of outpatients you normally take care of is cases/day

The number of inpatients you normally take care of is cases/day

The number of inpatients you normally take care of on night duty is

cases/day

**Part 2: Factors associated with COD**

1. Do you know the ways that death certificate information can be used?

□ Yes, it used in/for .

□ No, I don’t

□ I am not sure

2. In the clinical practice, how accurately do you record the underlying cause of death (COD) on the death certificate?

□ Absolutely correct

□Quite correct

□ Fairly correct

□ Absolutely incorrect

□ I am not sure

3. Which factors influence the quality and accuracy with which you complete a death certificate? (you can select more than 1 answer)

□ You do not know the appropriate underlying COD

□ You encounter an extenuating circumstance, like a family member of the deceased that requests that you alter the COD in order to conceal the fact that the patient had HIV infection

□ You have a high volume workload

□ There is inadequate patient medical data and patient history in the patient’s medical record

□ Other reasons…………………………………………………………………….

4. Do you think there are some differences between COD and mode of death (MOD)?

□ Yes, they are totally different (Please provide details and examples ……………………………………………………….…………………………….)

□ Yes, but sometimes I cannot definitely distinguish between them (Please provide details and examples …………………………………………….…………………………….)

□ No, but I am not sure (Please provide details and examples ……………………………………………………….…………………………….)

□ I do not know

**Part 3. Educational and supporting systems**

1. When you were a medical student, did you receive training in cause of death (COD) and death certificate (DC) documentation? If so, how many hours training did you receive and how were you trained?

□ Yes, ……………………… hr./all medical curriculum, by..

□ Self-learning □ Workshop

□ Lecture □ In a real-life setting

□ No

2. In your workplace, was training in COD and DC provided before you began practicing?

□ Yes (You can select more than one of the following)

□ Self-learning □ Workshop

□ Lecture □ In real-life setting with supervision

□ No

**3. About your experience in COD coding**

3.1 Before becoming a general practitioner, how many cases per month were you given the opportunity to complete the COD?

□ Never □ 1-5 cases □ 6-10 cases □ more than 10 cases

3.2 Since becoming a general practitioner and in real-life practice, in how many cases per month do you conclude the COD?

□ Never □ 1-5 cases □ 6-10 cases □ more than 10 cases

4. **About the system for documenting death certificate in your workplace**

**Office hours**

4.1 Who is the person responsible for completing death certificates?

□ Medical staff who takes care of the patient

□ Intern/general practitioner who takes care of the patient

□ Clinical year medical student who takes care of the patient

□ Not sure, it depends

□ Other – please provide details…………………….

4.2 Who is the person responsible for writing the death summary in discharge note documents and in the medical record?

□ Medical staff who takes care of the patient

□ Intern/general practitioner who takes care of the patient

□ Clinical year medical student who takes care of the patient

□ Not sure, it depends

□ Other – please provide details…………………….

4.3 Is the person in 4.1 and 4.2 most often the same person?

□ Yes □ No

□ Not sure, it depends on the situation

**After hours**

5.1 Who is the person responsible for completing the death certificate?

□ Medical staff who takes care of the patient

□ Intern/general practitioner who takes care of the patient

□ On-duty general practitioner or staff

□ Clinical year medical student who takes care of the patient

□ Not sure, it depends

□ Other – please provide details…………………….

5.2 Who is the person responsible for writing the death summary in discharge note documents and in the medical record?

□ Medical staff who take care of the patient

□ Intern/general practitioner who takes care of the patient

□ On-duty general practitioner or staff

□ Clinical year medical student who takes care of the patient

□ Not sure, it depends

□ Other – please provide details…………………….

5.3 Is the person in 5.1 and 5.2 most often the same person?

□ Yes □ No

□ Not sure, it depends on the situation
